# Tumor-suppressive role of Smad ubiquitination regulatory factor 2 in patients with colorectal cancer

**DOI:** 10.1038/s41598-022-09390-8

**Published:** 2022-03-31

**Authors:** Nami Sato, Nozomu Sakai, Katsunori Furukawa, Tsukasa Takayashiki, Satoshi Kuboki, Shigetsugu Takano, Gaku Ohira, Hideaki Miyauchi, Hisahiro Matsubara, Masayuki Ohtsuka

**Affiliations:** 1 Department of General Surgery, Graduate School of Medicine, Chiba University, 1-8-1 Inohana, Chuo-ku, Chiba, 260-8670 Japan; 2 Department of Frontier Surgery, Graduate School of Medicine, Chiba University, 1-8-1 Inohana, Chuo-ku, Chiba, 260-8670 Japan

**Keywords:** Cancer, Metastasis

## Abstract

Smad ubiquitination regulatory factor 2 (Smurf2) plays various roles in cancer progression. However, the correlation between Smurf2 and clinical outcomes has not been determined in patients diagnosed with colorectal cancer and colorectal liver metastases. We analyzed 66 patients with colorectal cancer who developed liver metastases. Smurf2 expression was assessed using immunohistochemical analysis of primary and metastatic liver tumors. High Smurf2 expression in both primary and metastatic tumors was significantly associated with longer overall survival time and time to surgical failure. Multivariate analyses revealed that low Smurf2 expression in primary tumors was an independent predictor of poor prognosis. In vitro experiments using colon cancer cell lines demonstrated that short interfering RNA knockdown of Smurf2 increased cell migration and tumor sphere formation. Western blot analyses revealed that Smurf2 knockdown increased the protein expression of epithelial cell adhesion molecule (EpCAM). Thus, in summary, high Smurf2 expression in cancer cells was found to be an independent predictor of better prognosis in patients with primary colorectal cancer and consequent liver metastases. The tumor-suppressive role of Smurf2 was found to be associated with cell migration and EpCAM expression; hence, Smurf2 can be considered a positive biomarker of cancer stem cell-like properties.

## Introduction

Colorectal cancer (CRC) is a leading cause of cancer-related death^1^. Distant metastasis is a strong factor marking poor prognosis in patients with CRC. Previous studies have demonstrated that a total of 25–30% of CRC patients were also diagnosed with liver metastasis^2–4^. Therefore, an accurate preoperative diagnosis and an appropriate treatment plan are essential to obtain better prognoses in patients with CRC.

Protein degradation mediated by the ubiquitin/proteasome system (UPS) controls various biological functions and is critical for maintaining homeostasis^5^. Additionally, the UPS plays important roles in several cancers^5,6^. Smad ubiquitination regulatory factor 2 (Smurf2) is a member of the homologues to E6-AP carboxyl terminus (HECT) family of E3 ubiquitin ligases. This E3 ligase was initially identified as a regulatory factor of transforming growth factor beta (TGF-β) signal transduction^7^. Since the identification of Smurf2, its various roles have been explored not only as a regulator of TGF-β but also as a direct regulator of the cell cycle and cancer development^8^. Although considerable evidence demonstrating the involvement of Smurf2 in cancer biology has been accumulated, the role of Smurf2 remains controversial. A previous study demonstrated the tumor-promoting role of Smurf2^9,10^, whereas other studies have demonstrated the tumor-suppressive role of Smurf2^11–14^. Accordingly, the role of Smurf2 in cancer biology seems to be “context-dependent”. Some studies have explored the tumor-suppressive role of Smurf2 and the corresponding mechanisms in the progression of CRC using a cell line and/or an animal model. Gao et al. demonstrated the tumor-suppressive role of Smurf2 using in vitro and in vivo studies. Additionally, the tumor-suppressive role of Smurf2 was reported to be associated with the degradation of YY1 and downregulation of SENP1/c-myc^15^. Yu et al. demonstrated that Smurf2 suppressed CRC cell proliferation and tumorigenesis through an interaction with sirtuin 1: Smurf2 depletion leads to sirtuin 1 upregulation and induces the tumor formation and growth of CRC in vitro and *in vivo*^12^. Li et al. demonstrated that Smurf2 reduces aerobic glycolysis and cell proliferation by promoting ChREBP ubiquitination and degradation via the proteasome pathway in CRC cells^16^. Pu et al. demonstrated that Smurf2 inhibited cell growth and metastasis in colon cancer and that Smurf2 regulation was involved in the anticancer effects of schisandrin B in both in vitro and in vivo models^17^. In contrast to the above studies that demonstrated the various mechanisms of Smurf2 in CRC using cell lines and/or animal models, one study demonstrated that Smurf2 expression was upregulated in CRC specimens and revealed that high Smurf2 expression was associated with impaired overall survival in microsatellite stable CRC, but not in microsatellite instable CRC^18^. Although this study assessed the association between Smurf2 expression and clinical outcomes, it only assessed primary CRC. No studies have yet comprehensively evaluated the role of Smurf2 in primary CRC and the corresponding liver metastases. Therefore, the aim of the current study was to evaluate the expression of Smurf2 in CRC and the corresponding colorectal liver metastases as well as its correlation with patient clinical outcomes. Furthermore, the molecular mechanisms underlying the clinical results reported herein were explored.

## Methods

### Patients and human tissue samples

CRC and corresponding liver metastasis tissues were obtained from 66 consecutive patients who underwent surgical resection for primary CRC and liver metastases at Chiba University Hospital (Chiba, Japan) between January 2005 and December 2014. Patients who underwent two-stage hepatectomy and primary tumor resection at other hospitals were excluded. Patients with synchronous liver metastases initially underwent primary tumor resection. As a control group, tissues were obtained from 60 consecutive patients with stage II or III CRC who did not develop distant metastases. These patients underwent surgical resection of the primary CRC between 2012 and 2014. The ethics committee of Chiba University, Graduate School of Medicine (Chiba, Japan) approved the protocol of the present study (approval number: 2405). The study protocol conformed to the provisions of the Declaration of Helsinki. Written informed consent was obtained from each patient before surgery. The clinical samples and their background information were obtained from the same database as in our previous study^19^.

### Indication criteria for surgical resection of metastatic tumors

The indications for resection of colorectal metastases were as follows: (1) curative resection of the primary tumor is possible or has already been performed; (2) curative resection of metastases is possible; (3) preservation of the physiological functions of the remaining tissue is possible (e.g., ≥ 40% of the total liver volume). The criteria are same with our previous study^19^.

### Immunohistochemistry (IHC)

Formalin-fixed paraffin-embedded tissue samples were cut into 4-µm-thick slices and deparaffinized. In the IHC for Smurf2, antigen retrieval was performed by microwaving in citric acid buffer (0.01 mol/L, pH 6.0) for 25 min. Subsequently, endogenous peroxidase activity was blocked using 3% hydrogen peroxide in methanol for 15 min. Non-specific proteins were blocked using 5% bovine serum albumin for 10 min. Following protein blocking, the slides were incubated at 4 °C overnight with the anti-Smurf2 monoclonal antibody (1:50 dilution; cat. no. sc-393848; Santa Cruz Biotechnology, Inc., Dallas, TX, USA). Counterstaining was performed with hematoxylin before dehydration, penetration, and mounting. The protocol of IHC is described in our previous study^19^.

### Immunohistochemical evaluation of Smurf2

Using an inverted microscope (BX40; Olympus Corporation, Tokyo, Japan), the expression levels of Smurf2 were evaluated independently by two investigators accompanied by a pathologist, all of whom were blinded to any clinical information. The intensity of tumor cell staining was scored as follows: 0, negative staining; 1, weak staining; 2, moderate staining; and 3, strong staining. Patients with a score of 0 or 1 were classified as having low expression, and those with scores of 2 or 3 were classified as having high expression. The immunohistochemical evaluations were performed after establishing an inter-observer consensus using samples from preliminary experiments.

### Human colon cancer cell lines and culture conditions

The human colon cancer cell line DLD-1 and the human colon cancer lymph node metastasis cell line SW620 were purchased from the American Type Culture Collection (USA). The DLD-1 cell line was cultured in RPMI 1640 medium (Gibco, Grand Island, NY, USA) supplemented with 10% fetal bovine serum (FBS) (Thermo Fisher Scientific, Inc., Waltham, MA, USA). The SW620 cell line was cultured in Leibovitz’s 15 medium (Gibco) supplemented with 10% FBS (Thermo Fisher Scientific, Inc.).

### Western blot analysis

Proteins were extracted from the cultured cells using RIPA buffer (Sigma-Aldrich, St. Louis, MO, USA). Each protein sample was lysed in a buffer (Laemmli Sample Buffer; Bio-Rad Laboratories, Inc., Richmond, CA, USA) containing 5% 2-mercaptoethanol and incubated at 97 °C for 10 min. After measuring the protein concentration of each sample using the Pierce™ BCA Protein Assay kit (Thermo Fisher Scientific, Inc.), 10 µg of protein was separated by electrophoresis on 5-12.5% XV PANTERA Gels (DRC, Tokyo, Japan) and transferred onto a membrane (PerkinElmer, Inc., Waltham, MA, USA). The membranes were blocked in 5% skim milk diluted with 0.1% Tris-buffered saline with Tween-20 at room temperature (15–25 °C) for 60 min. The membranes were then incubated at 4 °C overnight with the following primary antibodies: anti-Smurf2 polyclonal antibody (1:2,000 dilution; cat. no. ab94483; Abcam plc), anti-EpCAM polyclonal antibody (1:1,000 dilution; cat. no. HPA026761; Sigma-Aldrich) and anti-β-actin monoclonal antibody (1:5,000 dilution; cat. no. 5125; Cell Signaling Technology Inc., Beverly, MA, USA). Subsequently, the membranes were incubated in blocking buffer at room temperature (15–25 °C) for 60 min with anti-rabbit IgG horseradish peroxidase secondary antibody (1:2,000 dilution; cat. No. Sc-2301; Santa Cruz Biotechnology). The membranes were then incubated with an enhanced chemiluminescence detection reagent (Chemi-Lumi One Ultra; Nacalai Tesque, Inc., Kyoto, Japan) and developed with an LAS-4000UV mini luminescent image analyzer (Fujifilm, Tokyo, Japan). Band intensities were quantified by densitometric analysis using ImageJ software version 1.51 (National Institutes of Health, Bethesda, MD, USA) and were used to calculate the relative protein level normalized to β-actin. The protocol of western blot is described in our previous study^19^.

### Short interfering RNA (siRNA) transfection

The double-stranded siRNAs used to knock down Smurf2 expression were as follows: siSMURF2, Stealth siRNA (cat. no. HSS127687 and HSS127688; Thermo Fisher Scientific, Inc.). Negative control siRNA (AllStars negative control siRNA; Qiagen) was used as the control for all siRNA experiments. These siRNAs (final concentration, 2 nmol/L) were transfected into DLD-1 and SW620 cells using Lipofectamine™ RNAiMAX transfection reagent (Invitrogen; Thermo Fisher Scientific, Inc.) as previously described^19^. These cells were used for subsequent assays, 24 h post-transfection.

### Wound-healing assay

A wound-healing assay was conducted to assess cell migration ability using Culture-Insert 2 well in a 35-mm μ-dish (ibidi GmbH, Martinsried, Germany). DLD-1 and SW620 cells transfected with siSmurf2s or siControl were seeded at a density of 3.0 × 10^4^ cells/well and 1.0 × 10^5^ cells/well, respectively, and were cultured for 24 h. Cells were pre-treated with 5 µg/mL mitomycin C at 2 h prior to removal of the Culture-Insert. After removing the Culture-Insert, the wells were filled with the appropriate medium and allowed to heal for 24 h for DLD-1 and for 72 h for SW620. The open wound area was measured under a microscope at 50 × magnification.

### Cell proliferation assay

Cell proliferation was examined using CCK-8 (Dojindo Molecular Technologies, Inc., Kumamoto, Japan) as previously described^19^. The DLD-1 and SW620 cells transfected with siSmurf2s or siControl were seeded at a density of 1,000 cells/well in 96-well plates. CCK-8 (10 μL/well) solution was added to measure cell viability at 0, 24, 48, 72, and 96 h. After 2 h of incubation, the absorbance of each well was measured at 450 nm.

### Cell invasion assay

Cell Biolabs CytoSelect™ 24-well cell invasion assay kits (Cell Biolabs, San Diego, CA, USA), utilizing basement membrane-coated inserts, were used as previously described^19^. Briefly, DLD-1 and SW620 cells transfected with siSmurf2s or siControl were suspended in serum-free medium. Following overnight starvation, the cells were seeded at a density of 1.0 × 10^5^ cells/well in the upper chamber and incubated with the medium in the lower chamber for 48 h. The invasive cells passing through the basement membrane layer were stained, and the absorbance of each well was measured at 560 nm after extraction.

### Sphere formation assay

Tumor sphere formation assays were carried out as previously described^19^ with minor modifications. The DLD‐1 and SW620 cells transfected with siRNA or siControl were seeded in 96‐well ultra‐low attachment plates (Corning Inc., New York, NY, USA) at 15 cells/well and cultured in a sphere medium comprising DMEM‐F12 (1:1) medium (Gibco, Grand Island, NY, USA) containing 20 ng/mL human epidermal growth factor (BD biosciences, San Jose, CA, USA), 20 ng/mL human basic fibroblast growth factor (Invitrogen; Thermo Fisher Scientific, Inc.), 1% B27 Supplement (Invitrogen), 1% N2 Supplement (Invitrogen), 100 μM beta-ME (Bio-Rad Laboratories, Inc., Richmond, CA, USA), 1% Non-essential AA (Invitrogen), and 1% penicillin/streptomycin. After incubation for 7 d at 37 °C, the cells were evaluated and the number of spheres was counted using an inverted microscope (Axio Observer Z1; Carl Zeiss, Oberkochen, Germany). DLD1 with a diameter larger than 50 μm and SW620 with a diameter larger than 25 μm were counted as spheres. The sphere formation rate was calculated as scored sphere number/total plating cells.

### Statistical analysis

The correlations between Smurf2 staining and patient characteristics were evaluated using the χ^2^ test, Student’s t-test, or Mann–Whitney U-test, as appropriate. Survival rates were calculated using the Kaplan–Meier analyses and assessed using the log-rank test. Survival data were evaluated using the univariate and multivariate Cox proportional regression analyses. When analyzing the correlation between Smurf2 expression in the primary tumor and long-term outcomes, overall survival (OS), time to liver metastases, and time to surgical failure (TSF) were calculated from the date of primary tumor resection. TSF is defined as the interval between the time of initial surgery to the time of the first unresectable recurrence or death^20^. When analyzing the correlation between Smurf2 expression in liver metastases and the long-term outcome, OS, disease-free survival, and TSF were calculated from the date of initial hepatectomy. The in vitro experiments were performed at least three times independently, and data were analyzed using the Welch’s t-test and multivariate analysis of variance. Statistical significance was set at P < 0.05. Values are expressed as the mean ± standard error of the mean. The above series of statistical analyses were performed using JMP® PRO 13 software (SAS Institute Inc., Cary, NC, USA).

## Results

### High Smurf2 expression in the primary tumor is associated with better prognosis

**Figure 1:**
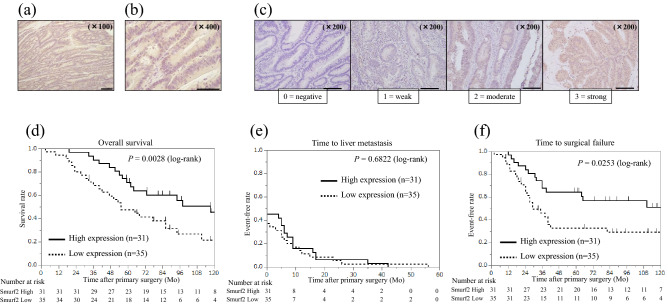
Smurf2 expression in primary CRC and the long-term outcome based on the Smurf2 expression. Immunohistochemistry analysis for Smad ubiquitination regulatory factor 2 (Smurf 2) expression in (**a**, **b**) primary colorectal cancer (CRC). Scale bar = 200 µm (a) and 100 µm (**b**). (**c**) Representative images of each score evaluating Smurf2 expression in primary CRC. Scale bar = 200 µm (**c**). The Kaplan–Meier analysis for (**d**) overall survival, (**e**) time to liver metastasis, and (**f**) time to surgical failure based on the Smad ubiquitination regulatory factor 2 (Smurf 2) expression in primary CRC.

Smurf2 protein expression was examined in primary tumors using IHC. Smurf2 was predominantly expressed in the cytoplasm of cancer cells in primary tumors (Fig. 1a,b). Furthermore, Smurf2 expression was evaluated based on the scoring system (Fig. 1c). In primary tumors, high Smurf2 expression was observed in 31 patients (47.0%), whereas low Smurf2 expression was observed in 35 patients (53.0%). Smurf2 expression profiles in primary tumors and clinicopathological features are shown in Table 1. Smurf2 expression was not associated with any clinicopathological features assessed in the present study. The Kaplan–Meier analysis showed that patients with high Smurf2 expression had a significantly better OS time and TSF after primary surgery than that of patients with low Smurf2 expression (P = 0.0028 and 0.0253, respectively; Fig. 1d,f). No significant difference in the time to liver metastasis was found between patients showing high and low Smurf2 expression (Fig. 1e).

**Table 1:** Clinicopathologic features of patients diagnosed with colorectal cancer showing high and low Smad ubiquitination regulatory factor 2 (Smurf2) expression.

	Smurf2 expression		*P* value
	High (n = 31)	Low (n = 35)	
Age at primary surgery (years), Median (range)	69 (50–82)	65 (46–81)	0.0952
Gender, Male/Female	21/10	23/12	1.0000
Site of tumor, Colon/Rectum	19/12	21/14	1.0000
Site of tumor, Right/Left/Rectum	6/13/12	13/8/14	0.1577
Degree of differentiation, tub, pap/por, muc	30/1	32/3	0.6161
Ly, 0–1/2–3	27/4	28/7	0.5207
V, 0–1/2–3	16/15	15/20	0.6217
T stage (8th edition), 1/2/3/4	1/0/17/13	0/2/17/16	0.5423
Lymph node metastasis, + / −	17/14	22/13	0.6177
CEA, median (range)	8 (1.4–5610)	6.9 (0.7–5700)	0.8786
CA19-9, median (range)	12 (0.1–702)	33.3 (0–16,200)	0.1561
KRAS mutation, Wild/Mutant	9/4	9/7	0.7021
Adjuvant chemotherapy, + / −	5/26	5/30	1.0000
Interval to liver metastasis (months), mean ± SD	6.0 ± 1.9	5.1 ± 1.8	0.7820
Timing of metastasis, Synchronous/metachronous	17/14	22/13	0.6177
Number of liver metastatic tumors, mean ± SD	2.9 ± 0.5	3.0 ± 0.5	0.8808
Number of liver metastatic tumors, Solitary/Multiple	10/21	13/22	0.7973
Size of liver metastatic tumors (cm), mean ± SD	3.2 ± 0.4	4.3 ± 0.4	0.0877
Site of liver metastasis, Unilateral/Bilateral	17/14	20/15	1.0000
Hepatectomy, Minor/Major	28/3	28/7	0.3138
H factor, H1/H2/H3	24/7/0	19/15/1	0.0914
Extrahepatic metastases, + / −	6/25	6/29	1.0000

CEA, carcinoembryonic antigen; CA19-9, carbohydrate antigen 19–9; KRAS, Kirsten rat sarcoma viral oncogene.

In the univariate analysis, the primary tumor site (colon vs. rectum, right-sided colon vs. left-sided colon), lymph node metastasis, surgical margin, H factor, and Smurf2 expression were correlated with OS. Among these, rectal cancer, positive lymph node metastasis, surgical margin (R1 and R2), and low Smurf2 expression were identified as independent poor prognosis factors (*P* = 0.0019, 0.0265, 0.0410, and 0.0370, respectively; Cox proportional hazards model; Table 2) in multivariate analyses. These data suggest that the higher expression of Smurf2 in primary tumors is associated with better prognosis.

**Table 2:** Prognostic factors for overall survival (OS) in colorectal cancer.

Prognostic factors	n	5-year OS rate (%)	Univariate P value	Multivariate HR (95% CI)	*P* value
Age at primary surgery, < 65/ ≥ 65	44/22	54.6/60.8	0.7742		
Sex, Male/Female	44/22	53.8/68.2	0.7217		
Site of primary tumor, Colon/Rectum	40/26	74.7/34.6	0.0022	0.30 (0.129–0.647)	0.0019
Site of primary tumor, Right/Left	19/47	78.6/51.1	0.0486	0.93 (0.339–2.541)	0.8864
CEA < 5/ ≥ 5	18/48	65.5/56.3	0.1631		
CA19-9 < 37/ ≥ 37	43/23	64.5/47.8	0.2476		
Degree of differentiation, tub, pap/por, muc	62/4	57.6/75.0	0.9649		
ly, 0–1/2–3	55/11	56.4/71.6	0.9228		
v, 0–1/2–3	31/35	71.0/47.5	0.1033		
T stage (8th edition), 1–3/4	37/29	64.9/50.6	0.2924		
Lymph node metastasis, − / +	27/39	69.7/51.3	0.0112	0.43 (0.195–0.909)	0.0265
KRAS mutation, Wild/Mutant	11/18	55.6/45.5	0.6377		
Adjuvant chemotherapy after primary surgery, − / +	56/10	60.2/50.0	0.3630		
Timing of liver metastasis, Synchronous/metachronous	27/39	53.9/65.8	0.2591		
Metastasis other than liver, − / +	54/12	62.5/41.7	0.1181		
Number of liver metastasis, Solitary/Multiple	23/43	69.1/53.5	0.1929		
Size of largest tumor (cm), < 5/ ≥ 5	53/13	65.6/46.2	0.3057		
Hepatectomy, Minor/Major	56/10	62.6/40.0	0.0687		
Surgical margin, R0/R1,R2	35/31	67.9/48.4	0.0287	0.49 (0.241–0.972)	0.0410
H factor, H1/H2-H3	43/23	71.6/34.8	0.0171	0.70 (0.327–1.548)	0.3713
Expression of Smurf 2 in primary tumor, High/Low	31/35	71.0/47.6	0.0265	0.47 (0.232–0.957)	0.0370

CEA, carcinoembryonic antigen; CA19-9, carbohydrate antigen 19–9; KRAS, Kirsten rat sarcoma viral oncogene.

### High Smurf2 expression in liver metastases is associated with better prognosis

**Figure 2:**
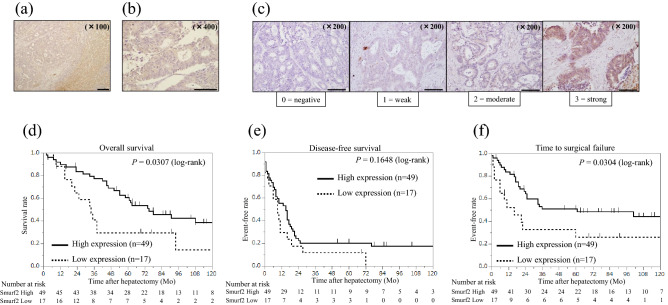
Smurf2 expression in colorectal liver metastases and the long-term outcome based on Smurf2 expression. Immunohistochemistry analysis for Smad ubiquitination regulatory factor 2 (Smurf 2) expression in (**a**, **b**) colorectal liver metastases. Scale bar = 200 µm (**a**) and 100 µm (**b**). (**c**) Representative images of each score evaluating Smurf2 expression in colorectal liver metastases. Scale bar = 200 µm (**c**). The Kaplan–Meier analysis for (**d**) overall survival, (**e**) disease-free survival, and (**f**) time to surgical failure based on the Smad ubiquitination regulatory factor 2 (Smurf 2) expression in metastatic liver tumors.

Smurf2 protein expression was examined in metastatic tumors in the liver using IHC. Smurf2 was predominantly expressed in the cytoplasm of cancer cells in liver metastases (Fig. 2a,b ). Further, Smurf2 expression was evaluated based on the scoring system (Fig. 2c). Clinicopathological features were compared between high and low levels of Smurf2 expression in liver metastases. As shown in Table 3, high Smurf2 expression was observed in 49 patients (74.2%), whereas low Smurf2 expression was observed in 17 patients (25.8%). Smurf2 expression was not associated with any clinicopathological features assessed in the present study. The Kaplan–Meier analysis showed that patients with high Smurf2 expression in liver metastases had significantly better OS time and TSF after hepatectomy than that of patients with low Smurf2 expression (P = 0.0307 and 0.0304, respectively; Fig. 2d,f). However, no significant difference in the disease-free survival (DFS) time after hepatectomy was found between patients showing high and low Smurf2 expression (Fig. 2e). These data suggest that higher Smurf2 expression in liver metastases is associated with better prognosis.

**Table 3:** Clinicopathologic features of patients diagnosed with colorectal liver metastases showing high and low Smad ubiquitination regulatory factor 2 (Smurf2) expression.

	Smurf2 expression		*P* value
	High (n = 49)	Low (n = 17)	
Age at primary surgery (years), Median (range)	68 (46–84)	69 (50–79)	0.9356
Gender, Male/Female	32/17	12/5	0.7726
Site of tumor, Colon/Rectum	31/18	9/8	0.5668
Site of tumor, Right/Left/Rectum	15/16/18	4/5/8	0.7397
Degree of differentiation, tub, pap/por, muc	47/2	15/2	0.2714
Ly, 0–1/2–3	40 / 9	15 / 2	0.7145
V, 0–1/2–3	24/25	7/10	0.7786
T stage (8^th^ edition), 1/2/3/4	0/1/25/23	1/1/9/6	0.2342
Lymph node metastasis, + / −	29/20	10/7	1.0000
CEA before hepatectomy, median (range)	50.7 ± 170.0	947.2 ± 288.6	0.0094
CA19-9, median (range)	129.9 ± 497.7	1825.8 ± 845.0	0.0886
KRAS mutation, Wild/Mutant	9/4	9/7	0.7021
Adjuvant chemotherapy, + / −	36/13	13/4	1.0000
Interval to liver metastasis (months), mean ± SD	5.6 ± 1.5	5.3 ± 2.6	0.9106
Timing of metastasis, Synchronous/metachronous	29/20	10/7	1.0000
Number of liver metastatic tumors, mean ± SD	3.0 ± 0.4	2.6 ± 0.7	0.6250
Number of liver metastatic tumors, Solitary/Multiple	17/32	6/11	1.0000
Size of liver metastatic tumors (cm), mean ± SD	3.6 ± 0.4	4.1 ± 0.6	0.4674
Site of liver metastasis, Unilateral/Bilateral	17/14	20/15	1.0000
Hepatectomy, Minor/Major	45/4	11/6	0.0143
H factor, H1/H2/H3	33/15/1	10/7/0	0.6689
Extrahepatic metastases, + / −	9/40	3/14	1.0000
Resection margin, R0/R1/R2	27/14/8	8/6/3	0.8372
Adjuvant chemotherapy after hepatectomy, + / −	36/13	13/4	1.0000
Recurrence after hepatectomy (all organs), + / −	38/11	16/1	0.1632
Intrahepatic recurrence after hepatectomy, + / − Number of tumors, mean ± SD	26/23 2.4 ± 0.5	10/7 3.8 ± 0.8	0.78100.1185
Recurrence in multiple organs, + / −	6/43	15/2	1.0000
Repeat resection (all organs), + / −	24/14	6/10	0.1332
Repeat hepatectomy, + / −	13/13	2/8	0.1422

CEA, carcinoembryonic antigen; CA19-9, carbohydrate antigen 19–9; KRAS, Kirsten rat sarcoma viral oncogene.

### Differential expression of Smurf2 in patients with liver metastases and patients with stage II or III CRC

When comparing Smurf2 expression in patients with liver metastases and patients with stage II or III CRC (i.e., without any distant metastases), high Smurf2 expression was observed in 47.0% of patients with liver metastases and 73.3% of patients with stage II or III CRC. Smurf2 expression was significantly higher in patients with stage II or III disease than in patients with liver metastases (*P* = 0.0035) (Supplementary Fig. 1). These data suggest that expression of Smurf2 in primary tumors is associated with CRC progression.

### Acceleration of tumor cell migration through Smurf2 knockdown

**Figure 3:**
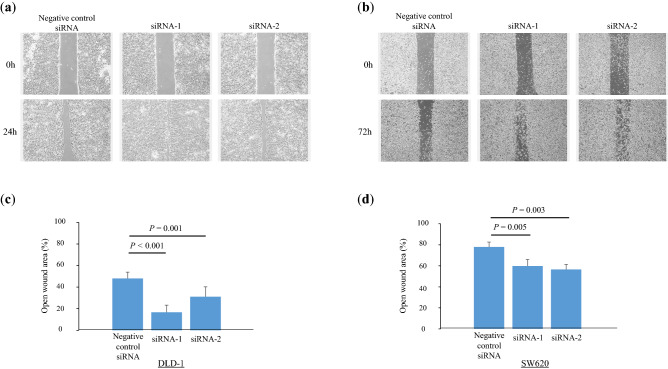
Wound-healing assay in colorectal cancer cells. (**a**, **b**) Representative images of wound healing in (**a**) human colon cancer cell line DLD-1 cells and (**b**) human colon cancer lymph node metastasis cell line SW620 cells treated with siControl and siSmurf2. (**c**, **d**) Open wound area was measured under a microscope at 50 × magnification in (**c**) human colon cancer cell line DLD-1 cells and (**d**) human colon cancer lymph node metastasis cell line SW620 cells.

The clinical data indicated that Smurf2 might play a tumor-suppressive role in CRC; therefore, in vitro experiments were performed to elucidate the molecular mechanisms by which Smurf2 regulates the behavior of CRC cells. The human colon cancer cell line DLD-1 and the human colon cancer lymph node metastasis cell line SW620 were used for these experiments. To assess the effects of Smurf2 on cell migration, we performed a wound-healing assay following the knockdown of Smurf2 using siRNAs. The wound-healing assay revealed that the knockdown of Smurf2 significantly prompted wound healing (i.e., increased cell migration) (DLD-1 siRNA1 *P* < 0.001, siRNA2 *P* = 0.001, SW620 siRNA1 *P* = 0.005, siRNA2 *P* = 0.003) (Fig. 3). These data suggest that the knockdown of Smurf2 expression accelerates tumor cell migration.

### Knockdown of Smurf2 expression did not alter tumor cell proliferation

To assess the effects of Smurf2 on tumor cell proliferation in vitro, we performed CCK-8 assays following the knockdown of Smurf2 using siRNAs. The CCK-8 assay revealed that the knockdown of Smurf2 did not alter the proliferation of the DLD-1 and SW620 cells (Supplementary Fig. 2). These data suggest that the knockdown of Smurf2 expression does not alter tumor cell proliferation.

### Knockdown of Smurf2 expression did not alter invasiveness of tumor cells

Cell invasiveness is an important property in the metastatic cascade of cancer; therefore, the effect of Smurf2 on cell invasiveness was assessed. Cell invasion assays revealed that the knockdown of Smurf2 did not alter the invasiveness of the DLD-1 and SW620 cells (Supplementary Fig. 3). These data suggest that the knockdown of Smurf2 expression does not alter the invasiveness of tumor cells.

### Knockdown of Smurf2 expression increased the EpCAM expression in colon cancer cells

**Figure 4:**
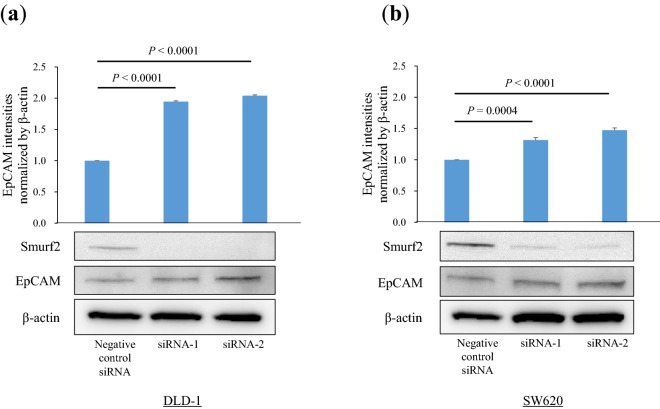
Western blot analyses for epithelial cell adhesion molecule (EpCAM) protein expression. Western blot analysis was performed to evaluate EpCAM protein expression in (**a**) human colon cancer cell line DLD-1 cells and (**b**) human colon cancer lymph node metastasis cell line SW620 cells treated with siControl and siSmurf2.

Western blot analyses were performed to evaluate the effects of Smurf2 expression on the protein expression levels of EpCAM. Western blot analyses revealed that Smurf2 knockdown significantly increased EpCAM protein expression in the DLD-1 and SW620 cells (DLD-1 siRNA1 P < 0.0001, siRNA2 P < 0.0001, SW620 siRNA1 P = 0.0004, siRNA2 P < 0.0001) (Fig. 4). These data suggest that the knockdown of Smurf2 expression increased the EpCAM expression in colon cancer cells.

### Knockdown of Smurf2 expression increased sphere formation

**Figure 5:**
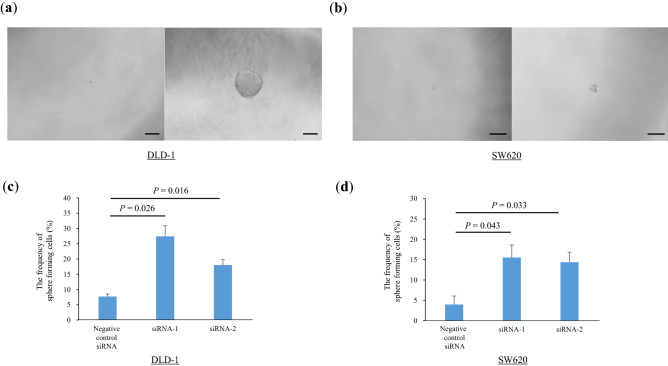
Sphere formation assay in colorectal cancer cells. (**a**, **b**) Representative pictures of sphere formation in DLD-1 (**a**) and SW620 (**b**) cells treated with siControl and siSmurf2. (**c**, **d**) The sphere formation rate were evaluated in DLD-1 (**c**) and SW620 (**d**) cells treated with siControl and siSmurf2.

To assess the effect of Smurf2 on the cancer stem cell-like properties, we performed a sphere formation assay. The sphere formation rate was significantly higher in siSmurf2 transfected cells than in the negative control siRNA-transfected cells (DLD-1 siRNA1 *P* = 0.026, siRNA2 *P* = 0.016, SW620 siRNA1 *P* = 0.043, siRNA2 *P* = 0.033) (Fig. 5). These data suggest that the knockdown of Smurf2 expression promotes sphere formation.

## Discussion

To the best of our knowledge, the present study is the first to demonstrate the prognostic impact of Smurf2 expression on clinical outcomes in patients with CRC and liver metastasis. Our results show that high Smurf2 expression in both primary CRC tumors and corresponding liver metastases was significantly associated with a better prognosis in patients with CRC and liver metastases. A novel finding in the present study is that Smurf2 modulates EpCAM expression. Using in vitro experiments, we demonstrated that the knockdown of Smurf2 expression accelerated cell migration, promoted sphere formation, and increased the expression of EpCAM, a cancer stem cell marker. These results indicate that Smurf2 acts as a negative regulator in CRC development and the metastatic cascade. These underlying mechanisms might be the cause of the early and unresectable recurrence of CRC, which ultimately leads to shorter survival times in patients with low Smurf2 expression.

EpCAM is a type-1 transmembrane glycoprotein that was initially considered a tumor antigen in colorectal carcinoma. Since its first description in 1979^21^, several studies have demonstrated the prognostic value of EpCAM in several cancers^22–27^. EpCAM is overexpressed in various cancers and the corresponding metastatic lesions^28^. Although the prognostic impact of EpCAM differs depending on the type of cancer, Seeber et al. demonstrated that high EpCAM expression in colon cancer cells was associated with aggressive tumor biology, causing multiple recurrences in the early phase following surgery in patients with CRC^26^. Our clinical data demonstrates that the prognosis was significantly worse in patients with low Smurf2 expression in both primary tumors and metastatic tumors compared to the prognosis of patients with high Smurf2 expression. Although our data may be too limited to draw a definitive conclusion, it is plausible that a decrease in Smurf2 expression could cause high EpCAM expression and consequently result in poor prognosis in patients with low Smurf2 expression. Additionally, our clinical data demonstrates that the TSF was significantly shorter in patients with low Smurf2 expression in both primary tumors and metastatic tumors. When considering the treatment strategy for CRC liver metastasis (CRLM), repeat resection for distant metastases, including liver and lung metastases, has been demonstrated to yield a survival outcome comparable to that of the initial hepatectomy^29–31^. Therefore, conventional recurrence-free survival is not necessarily associated with OS. However, TSF has been reported to be well correlated with OS in patients with CRLM^20^. Given these findings, low Smurf2 expression could be a cause of early and unresectable recurrence (i.e., shorter TSF), probably owing to the stem cell-like properties of cancer cells, and could be a good predictor of poor prognosis in patients. The mechanism(s) underlying shorter TSF in patients with low Smurf2 expression may include the involvement of Smurf2 in the stem cell-like properties of cancer cells. EpCAM expression has been associated with the stem cell-like properties of cancer cells^22^. Yamashita et al. demonstrated that there are distinctive EpCAM-positive cancer cell subpopulations in hepatocellular carcinoma, which have malignant potential for self-renewal, de-dedifferentiation, tumor initiation, invasiveness, and establishment of distant metastases^32,33^. Furthermore, positive EpCAM expression has been significantly associated with the development of distant metastases and shorter disease-free interval in patients with breast cancer^34^. Meanwhile, the sphere formation ability is also an important indicator of the stem cell-like properties of cancer cells^19^. Supporting the EpCAM expression results, Smurf2 knockdown was associated with the increase in the sphere formation rate, indicating that a decrease in Smurf2 expression increased the stem cell-like properties of cancer cells. Collectively, these data suggest that Smurf2 was associated with stem cell-like properties of colon cancer cells. These factors might contribute to the early and unresectable recurrence of CRC. The detailed molecular mechanisms by which Smurf2 modulates EpCAM expression and sphere-formation ability remain unclear. These findings need to be explored in future studies to expand the clinical implications of Smurf2.

Smurf2 expression was significantly lower in CRC with liver metastases than in stage II or III CRC. As shown in this study, low Smurf2 expression was associated with accelerated cell migration and an increase in stem cell-like properties and CTCs, which indicates the aggressive behavior of cancer cells. Therefore, Smurf2 might be an important modulator in CRC progression from primary tumor development to metastatic cascade and even recurrence following initial hepatectomy.

Our in vitro data demonstrate that Smurf2 knockdown significantly accelerated cancer cell migration, but not cell proliferation or invasion; the reasons for the latter remain unclear. Although previous studies have indicated that Smurf2 plays some roles in cell proliferation and invasion, reports of whether Smurf2 acts as a tumor-suppressor or tumor-promoter are varied^9,12–14,18^. Differences in the experimental models, cell line, or cell culture conditions might drive the variations in these results. Nonetheless, our in vitro results concerning cell proliferation were in line with our clinical results, demonstrating that Smurf2 expression was not significantly associated with tumor number or tumor size. Regarding the discrepancy in the association of Smurf2 with cell migration and with cell invasion, it may be considered that Smurf2 is not a potent modulator of cell motility. We speculate that these results may be consistent with the clinical results indicating that Smurf2 was not associated with DFS. In summary, the contribution of Smurf2 towards cell proliferation and invasion remains unknown and requires clarification in future studies.

The present study has several limitations. First, data were collected retrospectively from the database of a single institution. Therefore, the sample size was small, and the patients’ backgrounds were heterogeneous. Consequently, several prognostic factors were not randomized in the analyses of long-term outcomes. Second, the effect of perioperative chemotherapy was not assessed, because various regimens were used during the study period and the indication for perioperative chemotherapy was decided by physician’s preference in some cases. Therefore, it was difficult to evaluate the effect of systemic chemotherapy accurately. Third, we examined the effects of Smurf2 using loss-of-function experiments. However, gain-of-function experiments and in vivo experiments should be performed to verify our data and elucidate the role of Smurf2 in CRC progression more accurately.

In conclusion, high Smurf2 expression in cancer cells is an independent predictor of better prognosis in patients with primary CRC and corresponding liver metastases. The tumor-suppressive role of Smurf2 was found to be associated with cell migration and EpCAM expression, which is associated with stem cell-like properties of cancer cells. Further studies are warranted to verify our clinical data in a larger cohort and to explore the detailed molecular mechanisms underlying the role of Smurf2, which could ultimately lead to the development of therapeutic targets.

## Supplementary Information

Supplementary Information.

## Data Availability

The datasets generated during and/or analyzed during the current study are available from the corresponding author on reasonable request.
