# Supplementary material for: Tumor-suppressive role of Smad ubiquitination regulatory factor 2 in patients with colorectal cancer
Source: Sci Rep. 2022 Mar 31;12:5495. doi: 10.1038/s41598-022-09390-8 (PMC8971512; doi:10.1038/s41598-022-09390-8)
Supplement: Supplementary file 1 — Supplementary Information. [file 41598_2022_9390_MOESM1_ESM.pdf]

Supplementary figure 1

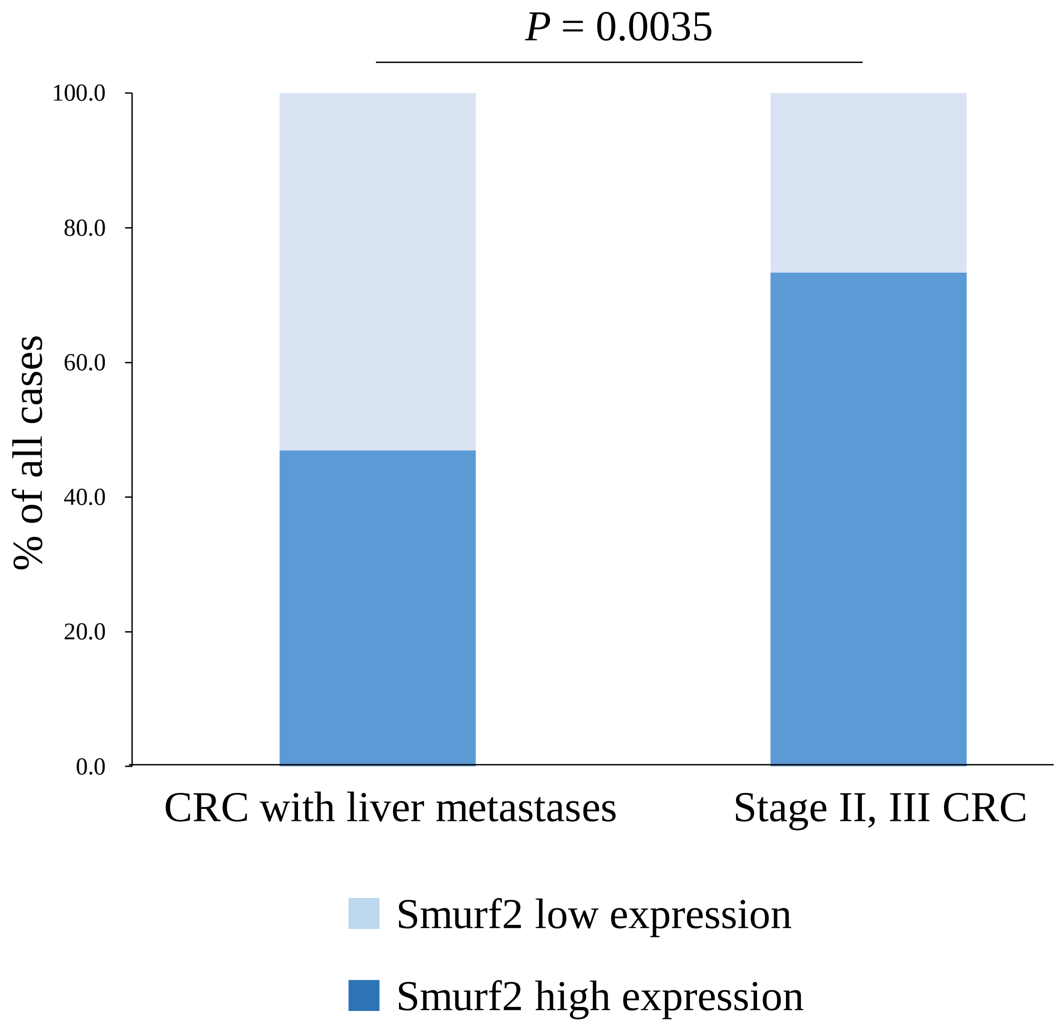

	High	Low
CRC with liver metastases	31	35
Stage II, III CRC	44	16

Smurf2 expression in CRC with liver metastases and Stage II, III CRC patients.
The association of Smad ubiquitination regulatory factor 2 (Smurf 2) expression in colorectal cancer (CRC) patients with liver metastases and patients with stage II or III CRC.

Supplementary figure 2

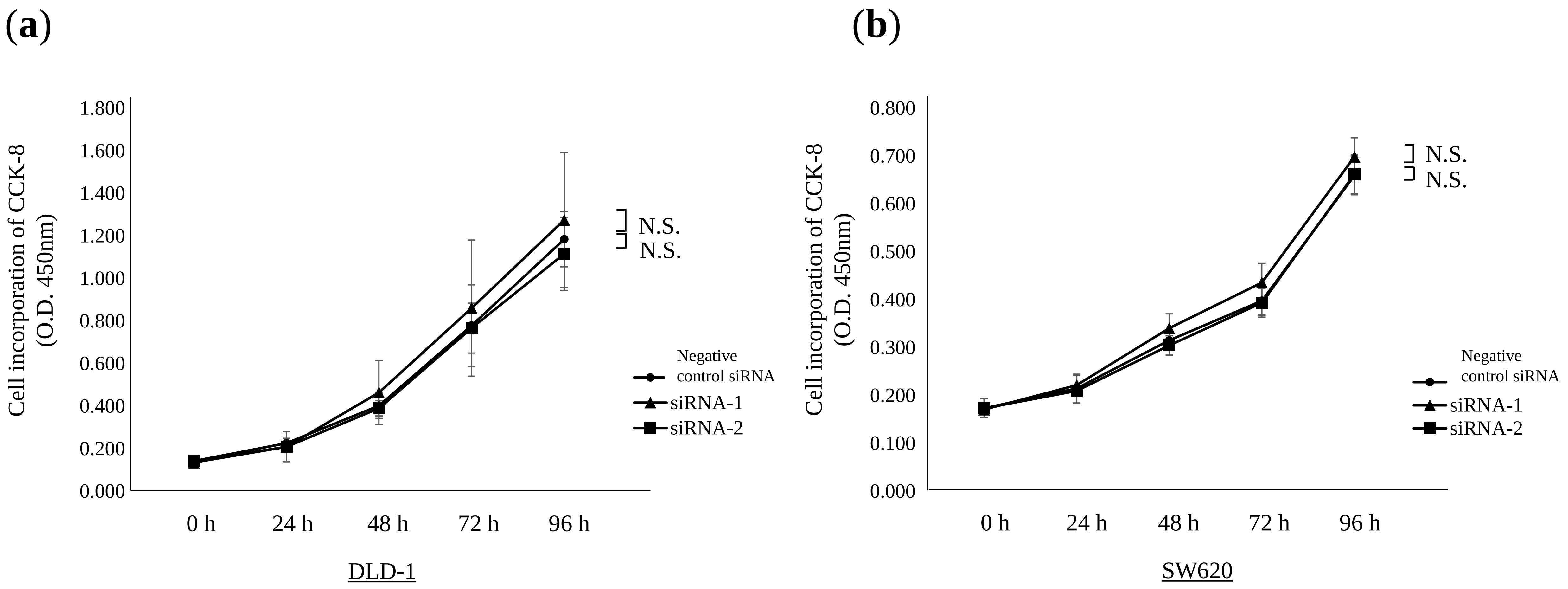

Cell proliferation assay using the CCK-8 assay in colorectal cancer cells.
The cell proliferation properties were assessed in human colon cancer cell line, DLD-1 (a), and human colon cancer lymph node metastasis cell line, SW620 (b). Cells were treated with negative control siRNA and siSmurf2.

Supplementary figure 3

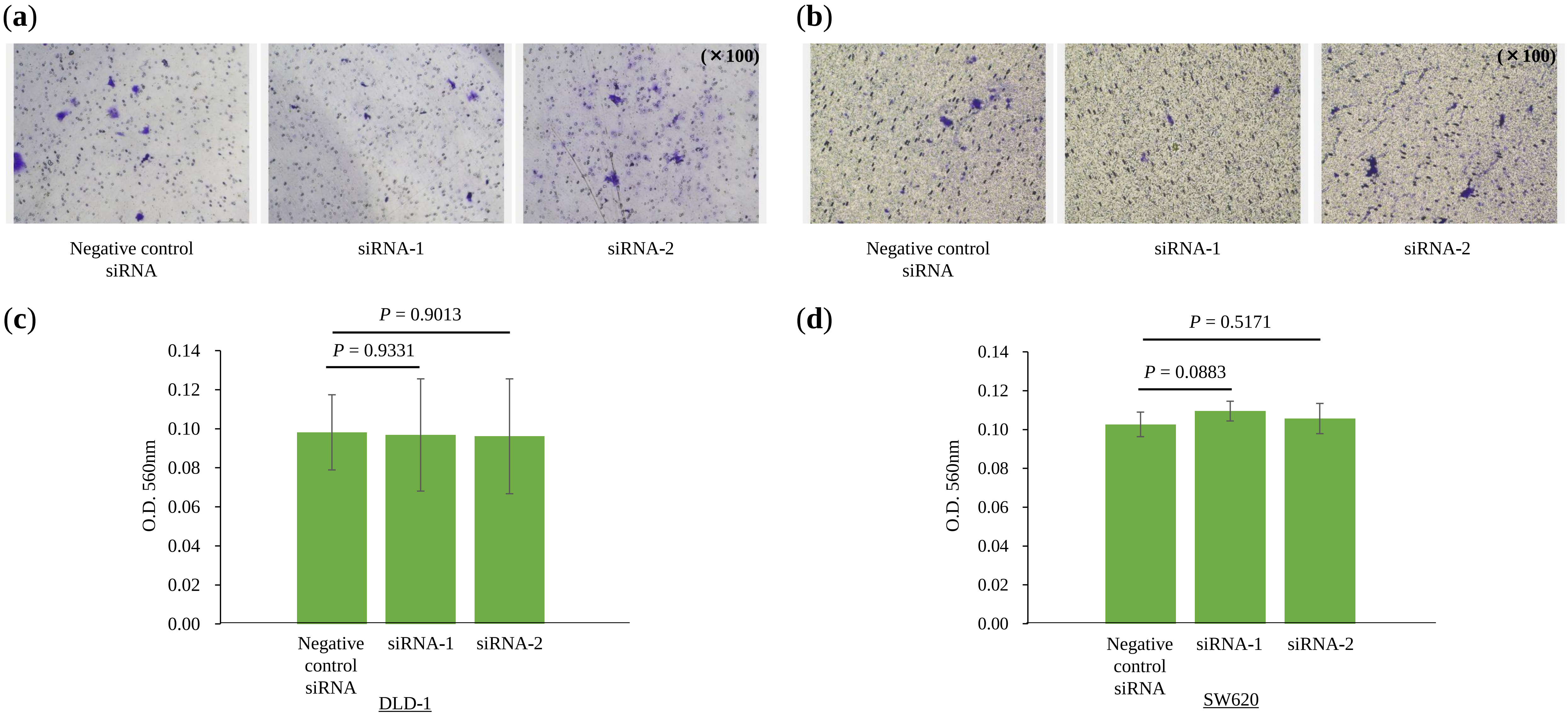

Cell invasion assay in colorectal cancer cells.

(a, b) Representative pictures of invasion assay in DLD-1 (a) and SW620 (b) cells treated with siControl and siSmurf2. The cell invasiveness were evaluated in DLD-1 (c) and SW620 (d) cells treated with negative control siRNA and siSmurf2.

Supplementary figure 4

(a) Smurf2

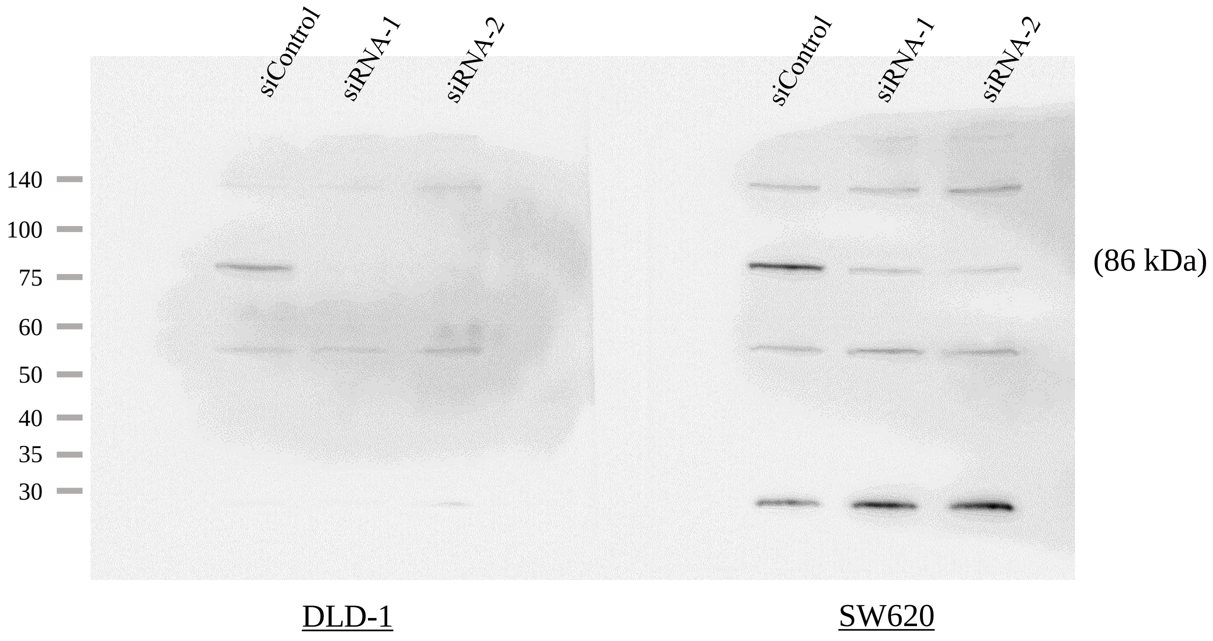

(b) EpCAM

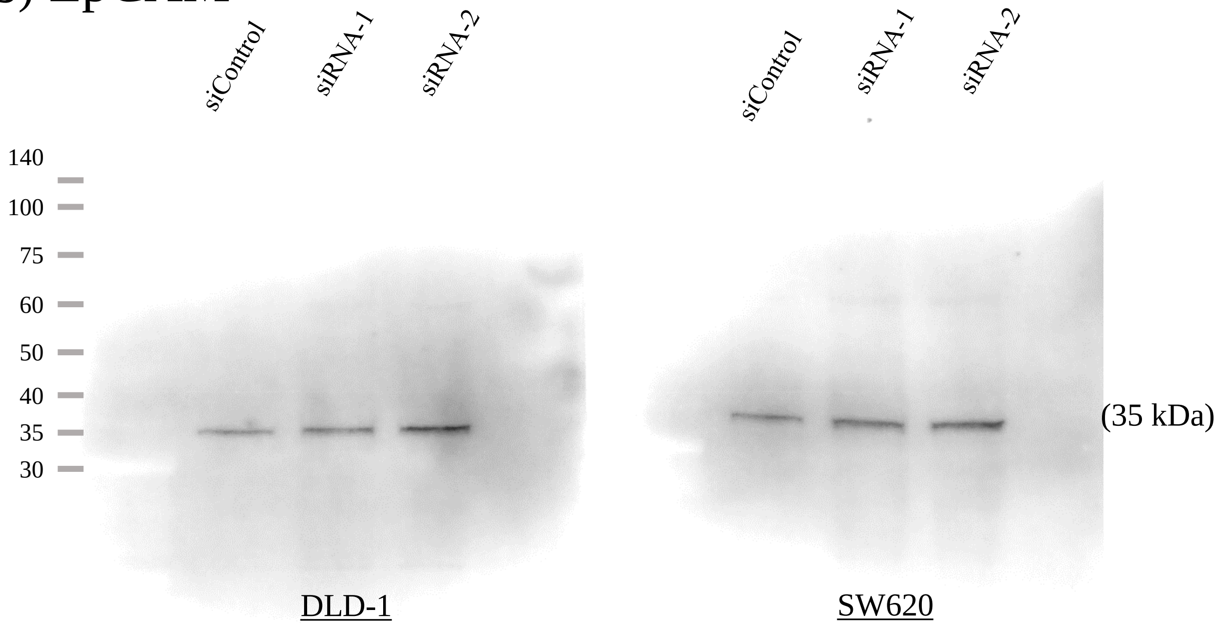

(c) β-actin

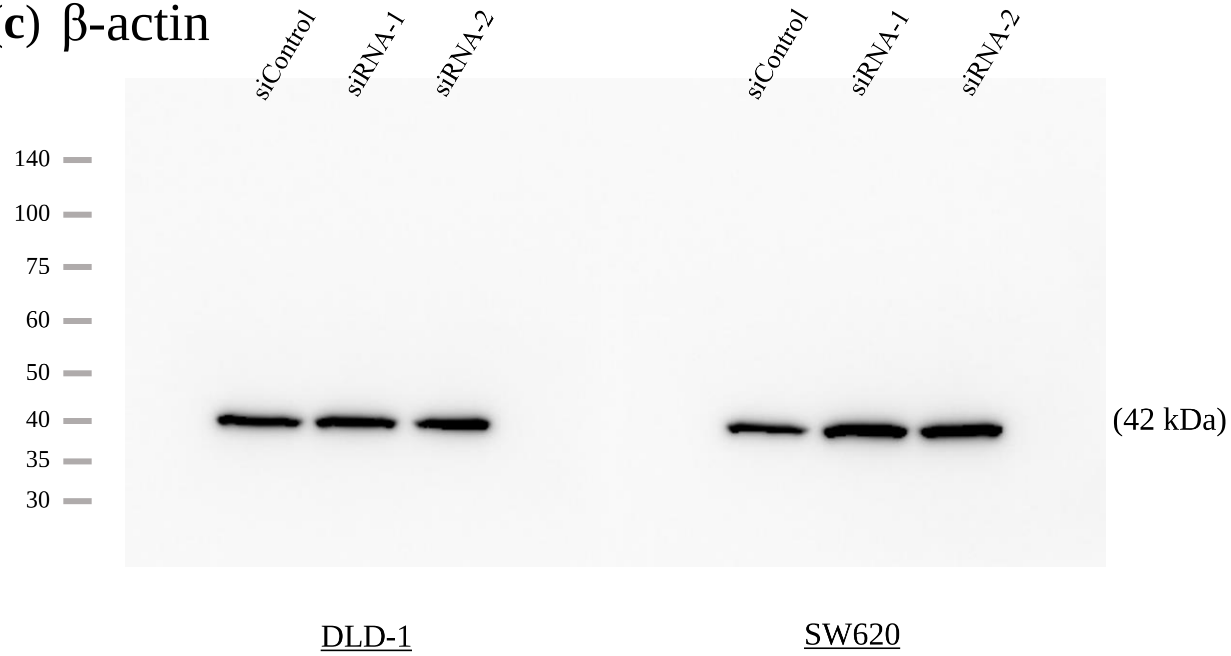

Western blot analyses for Smad ubiquitination regulatory factor 2 (Smurf 2), epithelial cell adhesion molecule (EpCAM), and β-actin protein.
Representative pictures of full-length gels and blots of Smurf2 (86kDa) (a), EpCAM (35kDa) (b) and β-actin (42kDa) (c).
Molecular weight markers are shown at the left.
